# Promoter DNA Methylation in GWAS-Identified Genes as Potential Functional Elements for Blood Pressure: An Observational and Mendelian Randomization Study

**DOI:** 10.3389/fgene.2021.791146

**Published:** 2022-01-11

**Authors:** Huan Zhang, Aili Wang, Tan Xu, Xingbo Mo, Yonghong Zhang

**Affiliations:** ^1^ Jiangsu Key Laboratory of Preventive and Translational Medicine for Geriatric Diseases, Medical College of Soochow University, Suzhou, China; ^2^ Department of Epidemiology, School of Public Health, Medical College of Soochow University, Suzhou, China; ^3^ Center for Genetic Epidemiology and Genomics, School of Public Health, Medical College of Soochow University, Suzhou, China

**Keywords:** promoter, methylation, genome-wide association study, risk factor, blood pressure

## Abstract

Genome-wide association studies have identified numerous genetic loci for blood pressure (BP). However, the relationships of functional elements inside these loci with BP are not fully understood. This study represented an effort to determine if promoter DNA methylations inside BP-associated loci were associated with BP.We conducted a cross-sectional study investigating the association between promoter DNA methylations of 10 candidate genes and BP in 1,241 Chinese individuals. Twenty-one genomic fragments in the CpG Islands were sequenced. The associations of methylation levels with BP and hypertension were assessed in regression models. Mendelian randomization (MR) analysis was then applied to find supporting evidence for the identified associations.A total of 413 DNA methylation sites were examined in an observational study. Methylation levels of 24 sites in *PRDM6*, *IGFBP3*, *SYT7*, *PDE3A*, *TBX2* and *C17orf82* were significantly associated with BP. Methylation levels of *PRDM6* and *SYT7* were significantly associated with hypertension. Methylation levels of five sites (including cg06713098) in *IGFBP3* were significantly associated with DBP. MR analysis found associations between the methylation levels of six CpG sites (cg06713098, cg14228300, cg23193639, cg21268650, cg10677697 and cg04812164) around the *IGFBP3* promoter and DBP. Methylation levels of cg14228300 and cg04812164 were associated with SBP. By further applying several MR methods we showed that the associations may not be due to pleiotropy. Association between *IGFBP3* mRNA levels in blood cells and BP was also found in MR analysis. This study identified promoter methylation as potential functional element for BP. The identified methylations may be involved in the regulatory pathway linking genetic variants to BP.

## Introduction

Hypertension, which affects more than one billion people worldwide, is identified as one of the most important causal risk factors for cardiovascular diseases (Verbanck et al., 2018; Zhu et al., 2018). In China, the prevalence of hypertension in adults (age >18) was estimated to be 23.2% (Wang et al., 2018), and about one third of people aged from 35 to 74 were hypertension (Lewington et al., 2016; Lu et al., 2017). About 2.5 million deaths are attributed to hypertension each year (Zhou et al., 2019). Therefore, hypertension is the most important public health problem in China.

As a complex trait, blood pressure (BP) is affected by the combinations of genetic and environmental factors. So far, the causal risk factors for hypertension were not fully understood. Genome-wide association studies (GWASs) have identified numerous genetic loci for BP (Ehret et al., 2011; Ehret et al., 2016; Evangelou et al., 2018). However, functional explanations for the genetic associations identified by GWASs were largely unknown. Determining functional elements inside the genetic loci were necessary for the explanations. Genetic variants could affect disease risks by altering gene expression and the regulations may always be indirect. Functional intermediate factors involved in the regulatory pathway linking genetic variants to BP probably exist and were not fully understood. The relationships between the functional elements inside the GWAS identified loci and BP need to be determined.

DNA methylation, which occurs primarily on CpG dinucleotide to control gene expression, is a key player in medicine (Dor and Cedar, 2018; Feinberg, 2018). DNA methylation has been identified as potential drug targets in the treatment of cardiovascular diseases (Byrne et al., 2014; Hu et al., 2017). Candidate gene and epigenome-wide association studies have strongly supported the importance of DNA methylation in the regulation and maintenance of BP levels for relevant biological insights, reliable biomarkers, and possible future therapeutics (Wang et al., 2018). Promoter DNA methylations most often leads to gene silencing by altering DNA structure and/or by inhibiting transcription factor binding. CpG Islands in 5′ regions also overlapped with promoters or enhancers (GeneHancer regulatory elements) (Fishilevich et al., 2017). Moreover, CpG sites may have long-range interactions with nearby promoters or enhancers. It is known that DNA methylations in long-range interactive promoters and enhancers can affect the long-range interactions and may be associated with the risk of disease (Park et al., 2017; Schoenfelder and Fraser, 2019). Therefore, promoter DNA methylations were key functional elements inside the genes.

**FIGURE 1:**
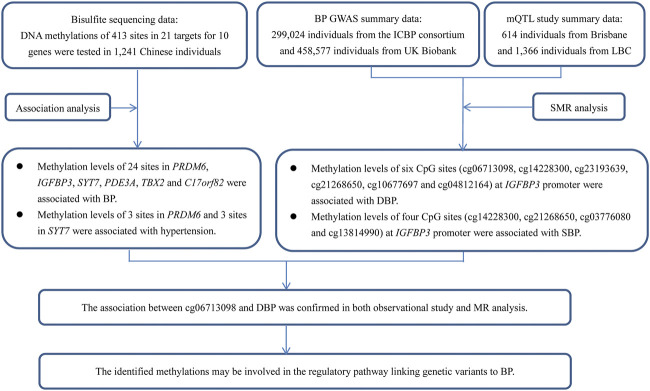
A flow chart showed the design of the present study. This study contained two parts of analyses, which aimed to find evidence to show the associations between DNA methylations and BP in genetic loci identified by GWAS. The first part was an observational study examining the associations between DNA methylations at 413 sites in 21 targets for 10 genes and BP in 1,241 Chinese individuals. The second part was an MR analysis using GWAS summary data from Europeans. The usage of summary data from Europeans can provide supporting evidence for the associations between the methylations and BP, because the causal associations between DNA methylations and BP may not be population specific, even though the data (i.e., genetic associations) used to find the evidence was population specific. Although little evidence was found in the MR analysis, some associations were validated, for example, site cg06713098 was associated with DBP in MR analysis. This site was tested and analyzed in the observational study (IGFBP3_2_113), and was significantly associated with DBP. Thus, the association between cg06713098 and DBP was confirmed in both observational study and MR analysis.

A *trans*-ancestry GWAS identified 12 genetic loci associated with BP in a large, multi-ancestry cohort (320,251 individuals of East Asian, European, and South Asian ancestry in total) (Kato et al., 2015). However, knowledge about the contribution of the identified genetic components to hypertension was vague at best. The sentinel SNPs in these 12 loci pointed to 20 candidate genes, including several genes involved in vascular smooth muscle and renal function. Sequence variants in these genes have a direct effect on DNA methylation levels in blood cells; moreover, some of the methylations were associated with BP among Europeans and South Asians, suggesting that DNA methylation may be involved in the regulatory pathway. Until now, however, the causal relationship of the methylation of these genes on BP regulation has not been determined. Evaluation of the effect of DNA methylation on hypertension risk will increase our understanding of the pathogenic molecular mechanisms and uncover new risk factors. In this study, we attempted to validate the relationships and uncover the potential molecular mechanisms by applying observational and Mendelian randomization (MR) analysis (Figure 1).

## Methods

### Cross-Sectional Study

#### Study Sample

We investigated the association between DNA methylation levels and BP and hypertension among Chinese Han individuals from the Suzhou Metabolic Syndrome Study (SMSS) (Kato et al., 2015). SMSS was an observational cohort study on the relationship between metabolic syndrome and components and cardiovascular disease events in six rural townships in of Suzhou in China’s Jiangsu province. A total of 12,461 individuals who had no evidence of end organ damage, including coronary heart disease, stroke, chronic renal disease and tumors, and signed informed consent were recruited to participate in SMSS. Longitudinal data were collected in every 2 years. We randomly selected 10% of individuals from the SMSS participants in the present study to test DNA methylation levels by sequencing technology and assess the associations between DNA methylation levels and BP. The included participants had no chronic kidney disease, a definite diagnosis of heart disease, acute infectious disease, chronic wasting disease, or serious liver disease. The basic characteristics about the overall cohort and the selected cohort were presented in Supplementary Table S1. Baseline blood samples were used in DNA methylation testing. The SMSS was approved by the Institutional Review Boards or Ethical Committees at Soochow University. Written consents were obtained from all study participants.

#### Data Collection

For all the participants, data on demographic information, lifestyle risk factors, family history of cardiovascular disease, and personal medical history were obtained using a standard questionnaire administered by trained staff. Three sitting consecutive BP measurements (30 s between each) were taken by trained staff using a standard mercury sphygmomanometer according to a standard protocol, after the subjects had been resting for 30 min. The first and fifth Korotkoff sounds were recorded as systolic BP (SBP) and diastolic BP (DBP), respectively. The mean of the three readings was used in the analysis. Hypertension was defined as SBP ≥140 mmHg and/or DBP ≥90 mmHg and/or use of antihypertensive medication in recent 2 weeks (James et al., 2014). Overnight fasting blood samples were obtained, plasma (serum) and white blood cell samples were isolated, and DNA was extracted from white blood cells for all the participants. The specimens were frozen at −80°C until laboratory testing. A modified hexokinase enzymatic method was applied to test fasting blood Glucose (FBG) levels. Total cholesterol (TC), High-density lipoprotein cholesterol (HDL-C) and triglycerides (TG) were analyzed enzymatically using a Beckman Synchron CX5 Delta Clinical System (Beckman Coulter, Inc., Fullerton, California, Unites States) with commercial reagents. Low-density lipoprotein cholesterol (LDL-C) levels were calculated using the Friedewald equation for participants who had less than 400 mg/dL TG.

#### DNA Methylation Sequencing

The current gold standard for the detection of DNA methylation is bisulfite sequencing. To measure the DNA methylation levels of genes in the 12 loci (Kato et al., 2015), targeted bisulfite sequencing (MethylTarget™) developed by Genesky Biotechnologies Inc. (Shanghai, China) were carried out as previously described (Zhu et al., 2019). Promoter sequences of 20 genes in the 12 loci were evaluated (Supplementary Table S2). Briefly, for each gene, CpG Islands adjacent to the promoter region (from 2 kilo bases upstream of the transcription start site to 1 kilo bases downstream of the first exon) were analyzed, and a genomic fragment from the CpG Island (named target) was determined and sequenced. Genomic DNA passed quality control (concentration ≥20 ng/μL, total DNA ≥ 1 μg, OD260/280 = 1.7–2.0, OD260/230 ≥ 1.8) was bisulfite converted using the EZ DNA Methylation-Gold Kit (ZYMO, CA, Unites States), and the targeted DNA sequences were amplified by PCR (Supplementary Table S3). The products were sequenced on an Illumina MiSeq benchtop sequencer (Illumina, CA, United States).

Based on these CpG Islands, 21 targets from CpG Islands of 10 genes were finally sequenced. Methylation level at each CpG site was calculated as the percentage of methylated cytosines over the total tested cytosines (reads). The average methylation level for a target was calculated by using methylation levels of all measured sites within the target. To test the reliability of methylation levels for each methylation site, 20 samples were randomly selected for duplicate detection. There was no significant difference between the two tests for the methylation levels of each of these sites.

#### Statistical Analysis

The differences in baseline risk factors between cases and controls were compared, using a Student’s *t*-test or Wilcoxon two sample test for continuous variables and *χ* ^2^ tests for categorical variables. Multiple regression model was performed to adjust for any potential confounders in the risk at baseline comparison. The methylation levels of each methylation site and gene in hypertensive cases and controls were assessed by two-tailed unpaired Student′s *t*-tests and multiple regression models adjusted for covariates; bar plots depict the means, and the error bars in the figures represent standard errors. For individuals being treated with BP-lowering medication, the following adjustments to the BP values were made before performing the regression analysis: SBP +15 mmHg and DBP +10 mmHg.

Linear regression models were used to assess the associations between methylation levels and BP. Logistic regression models were used to calculate Odds ratios (OR) and 95% confidence intervals (CI) for hypertension and ischemic stroke for per 1-percent increased with methylation levels. Potential covariates, age, BMI, TC, TG, HDL-C, LDL-C and FBG, were included in the multivariate models. We also performed sensitivity analyses to estimate the covariate contributions in the associations between DNA methylation levels and BP. All analyses were performed using the R language program (Version 3.5.0). The significance level was set at *p* < 1.0 × 10^–4^ for the methylation sites (≈0.05/413, Bonferroni correction). FDR adjustment was also evaluated in addition to Bonferroni post hoc analyses. All analyses were performed using the R language program (Version 3.5.0).

### Mendelian Randomization Analysis in Europeans

#### Study Design

In order to obtain supporting evidence for methylations identified in the observational study, we applied MR approaches to test the associations between methylations in the 10 genes and BP. Summary data-based MR (SMR) method can identify functionally relevant genes at loci identified in GWAS by integrating summary-level data from GWAS summary statistics and expression quantitative trait locus data (Pavlides et al., 2016; Zhu et al., 2016). In this research, we conducted SMR analysis to further clarify whether the detected methylation was associated with BP. The public summary data from large-scale GWAS and methylation quantitative trait loci (mQTL) meta-analysis studies was applied by the SMR method to verify the pleiotropic association in a very large sample size, which would contribute to increasing the statistical power.

In addition, we employed the inverse-variance weighted (IVW) MR (Burgess et al., 2013), the MR-Egger (Bowden et al., 2015), the MR pleiotropic residual sum and outlier (MR-PRESSO) (Verbanck et al., 2018) and the Causal Analysis Using Summary Effect estimates (CAUSE) (Morrison et al., 2020) methods to test for potential causal relationships between methylation and BP. The IVW MR and MR-Egger analyses were performed by using the MendelianRandomization R package (Yavorska and Burgess, 2017). MR-PRESSO and CAUSE were applied to account for horizontal pleiotropic effects. In addition, associations between mRNA (eQTL data) and circulating protein (pQTL data) levels and BP were also examined by the MR methods.

#### Data Resources

The BP GWAS (Evangelou et al., 2018) dataset comprised the summary statistics for the association between more than 7 million SNPs and SBP and DBP that were evaluated in 757,601 individuals, including 299,024 individuals from the ICBP consortium (Ehret et al., 2011; Wain et al., 2017) and 458,577 individuals from the United Kingdom Biobank (Sudlow et al., 2015). This dataset can be found at the GWAS Catalog (https://www.ebi.ac.uk/gwas/, accession number GCST006624 and GCST006630). The necessary data for SMR analysis, including the rs number, effect allele, other alleles, frequency, beta, standard error, *p* value and sample size for each SNP, was extracted from this dataset.

We obtained high quality QTL data from large studies. The mQTL summary data (in binary format: https://cnsgenomics.com/software/smr/#DataResource) were required for the SMR analysis as well (McRae et al., 2018). DNA methylation levels in blood cells were detected by the Illumina HumanMethylation450 BeadChips among 614 individuals from the Brisbane Systems Genetics Study and 1,366 individuals from the Lothian Birth Cohorts. Data from 88,712 DNA methylation probes were available. SNPs within 2 Mb distance from each methylation probe were included in *cis*-mQTL analysis. Methylation probes with at least one *cis*-mQTL signal at *p* < 5.0 × 10^–8^ were used in the MR analyses. This was the largest mQTL data available for SMR analysis at present.

The eQTL study is the study conducted by Westra et al., which is the largest eQTL meta-analysis so far in peripheral blood samples of 5,311 European healthy individuals (Westra et al., 2013). The summary data was available at https://cnsgenomics.com/software/smr/#eQTLsummarydata. The first pQTL study tested genome-wide associations between 509,946 SNPs and plasma levels of 1,124 proteins in blood samples of 1,000 individuals from the KORA study (http://metabolomics.helmholtz-muenchen.de/pgwas/index.php?task=download) (Suhre et al., 2017). The second pQTL study performed genome-wide testing of 10.6 million imputed autosomal variants against levels of 2,994 plasma proteins in 3,301 individuals of European descent from the INTERVAL study (Sun et al., 2018). The summary data was available at http://www.phpc.cam.ac.uk/ceu/proteins/.

#### Data Analysis

The SMR (version 0.712) with default parameters in a command line program, downloaded from cnsgenomics.com/software/smr/, was used to perform the analysis. The linkage disequilibrium (LD) correlation for SMR analysis was calculated using the Genotype data of HapMap r23 CEU as a reference panel. The pleiotropic associations identified in SMR analysis do not necessarily mean that methylation and BP were affected by the same underlying causal variant, as the association could possibly be due to the top associated *cis*-mQTL in LD with two distinct causal variants, one only affecting gene expression and the other only affecting BP (named heterogeneity). The heterogeneity in dependent instruments (HEIDI) was performed in the resulting association statistics to test for heterogeneity. *P*_HEIDI_>0.05 indicates that there was no significant heterogeneity of QTL signals, that is, there was a single causal variant in the locus.

Summary statistics for the associations between genome-wide SNPs and methylation levels of the concerning sites and expression levels of the concerning genes were extracted from the QTL datasets. In CAUSE analysis, genome-wide summary statistics were used to calculate the nuisance parameters. SNPs with *p* value less than 5.0 × 10^–4^ were selected to be potential instrumental variables. We clumped SNPs (LD *r* ^2^ < 0.01 within 10,000 kb) based on data from the Europeans from the 1,000 Genomes project using the “clump_data” function in the R package TwoSampleMR to select independent instrumental variables.

## Results

### Association Between Methylation Levels and BP

**FIGURE 2:**
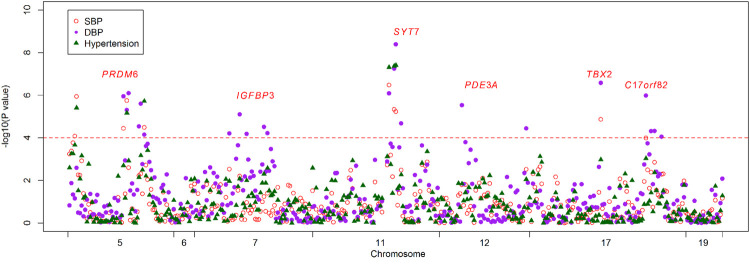
The association between DNA methylation and BP and hypertension. The observational study examined the associations between DNA methylation levels of the 413 tested sites and blood pressure (BP) and hypertension. The *x*-axis represents the chromosome positions. The *y*-axis shows the –log_10_ *P* values for the associations between methylation levels and SBP (red circles), DBP (purple dot) and hypertension (green triangles). Twenty-four sites at six genes achieved a threshold of 1.0 × 10^–4^ (red line).

Basic characteristics of the study participants were presented in Table 1. A total of 1,241 individuals were finally sequenced. We tested 413 DNA methylation sites in 21 targets for the 10 genes. These sites were in or around CpG Islands. By applying FDR adjustment in post hoc analysis (FDR < 0.05), the methylation levels of 20 and 72 sites were associated with SBP and DBP (Supplementary Tables S4 and S5), respectively. Methylation levels of 24 sites were significantly associated with SBP or DBP (*p* < 1.0 × 10^–4^). The significant sites were located in six genes, including *PRDM6*, *IGFBP3*, *SYT7*, *PDE3A*, *TBX2* and *C17orf82* (Figure 2). The top signals were located at the 5′ end of the *SYT7* gene, where the methylations were significantly associated with both SBP and DBP. Methylation levels of sites at the *PRDM6* and *TBX2* genes were also significantly associated with both SBP and DBP, while the methylation levels of sites in *IGFBP3*, *PDE3A* and *C17orf82* were associated only with DBP (Figure 2). In *IGFBP3*, five sites were found to be significantly associated with DBP (Table 2). According to the sensitivity analyses, the covariates did not substantially disturb the associations between DNA methylation levels and BP (Supplementary Tables S4 and S5).

**TABLE 1:** Characteristics of study participants.

Characteristics	Hypertensive cases (*n* = 485)	Controls (*n* = 756)	*p* Values	Adjusted *p* values
Age, year	66.51 ± 11.46	57.38 ± 11.02	1.56 × 10^–41^	9.97 × 10^–10^
Male, %	52.16	56.75	0.1135	0.508
Smokers, %	35.26	33.07	0.4268	0.975
Drinkers, %	26.60	26.46	0.9556	0.587
SBP, mmHg	150.47 ± 24.23	124.87 ± 17.55	1.16 × 10^–87^	1.27 × 10^–37^
DBP, mmHg	89.03 ± 12.68	75.56 ± 10.40	6.01 × 10^–80^	1.05 × 10^–25^
BMI, kg/m^2^	23.04 ± 3.59	21.64 ± 2.88	5.93 × 10^–14^	0.408
TC, mmol/L	4.80 ± 0.98	4.49 ± 0.90	1.03 × 10^–8^	0.949
TG, mmol/L	1.72 ± 1.23	1.36 ± 0.94	7.91 × 10^–9^	0.668
HDL-C, mmol/L	1.33 ± 0.33	1.37 ± 0.33	1.18 × 10^–2^	0.519
LDL-C, mmol/L	2.74 ± 0.77	2.52 ± 0.75	5.95 × 10^–7^	0.777
FBG, mmol/L	5.20 ± 1.07	4.96 ± 0.93	1.25 × 10^–4^	0.319

**TABLE 2:** Significant associations between *IGFBP3* methylation and DBP.

Sites	CHR	Position^a^	Crude beta	Crude *P*	FDR	Adjusted beta^b^	Adjusted *P* ^b^	FDR
IGFBP3_2_193	7	45960288	1.1089	6.16E−05	2.01E−03	0.8042	1.61E−03	2.37E−02
IGFBP3_2_155	7	45960326	0.8303	7.94E−06	4.25E−04	0.6579	1.20E−04	3.37E−03
IGFBP3_2_113	7	45960368	0.9384	6.51E−05	2.35E−03	0.7302	7.30E−04	1.89E−02
IGFBP3_2_49	7	45960432	0.6365	3.10E−05	6.53E−04	0.5179	2.29E−04	3.37E−03
IGFBP3_2_43	7	45960438	1.1663	6.03E−05	2.67E−03	0.9097	6.68E−04	1.89E−02

a The genomic position assembly was hg19.

b In linear regression models age, sex, smoking, BMI, total cholesterol levels and FBG, were adjusted.

At the target level, higher methylation level of target PRDM6_2 was associated with lower SBP levels (Table 3). Higher methylation levels of target C17orf82_3, HDAC9_1, IGFBP3_2, PRDM6_4, PRDM6_5 and SYT7_1 were associated with higher DBP levels (Table 3). Methylation levels of sites in *HDAC9* were not significantly associated with BP, but the methylation level of the target HDAC9_1 was significantly associated with DBP (Table 3). In different regression models adjusted for different covariates, the associations between methylation levels of these targets were significant (Table 4).

**TABLE 3:** The associations between methylation levels of the tested targets and BP and hypertension.

Targets	SBP				DBP				Hypertension			
	Beta	*p* value	Beta^a^	*p* value^a^	Beta	*p* value	Beta^a^	*p* value^a^	OR (95%CI)	*p* value	OR(95%CI)^a^	*p* value^a^
AMH_3	−0.0927	8.56E−01	0.0390	9.30E−01	0.1880	4.82E−01	0.1391	5.73E−01	1.00 (0.98, 1.02)	9.05E−01	1.00 (0.98, 1.02)	8.48E−01
C17orf82_2	−0.7521	5.00E−01	−0.8091	4.00E−01	0.0548	9.25E−01	0.1785	7.40E−01	0.98 (0.93, 1.03)	3.81E−01	0.98 (0.94, 1.02)	3.22E−01
C17orf82_3	1.3096	2.25E−02	0.9206	6.26E−02	1.0403	**5.24E−04**	0.9930	**3.24E−04**	1.02 (1.00, 1.05)	8.78E−02	1.01 (0.99, 1.04)	2.27E−01
C17orf82_5	−1.5594	2.14E−01	−1.1823	2.78E−01	0.0081	9.90E−01	0.0217	9.72E−01	0.98 (0.93, 1.04)	4.83E−01	0.99 (0.94, 1.04)	6.12E−01
HDAC9_1	1.3787	1.71E−02	1.1370	2.31E−02	1.1825	**8.60E−05**	0.8770	**1.67E−03**	1.03 (1.00, 1.05)	5.59E−02	1.02 (0.99, 1.04)	1.79E−01
IGFBP3_2	0.6775	1.76E−01	0.3122	4.70E−01	0.9563	**2.59E−04**	0.7357	**2.30E−03**	1.02 (1.00, 1.05)	3.14E−02	1.01 (1.00, 1.03)	1.36E−01
IGFBP3_3	−1.0828	4.65E−02	−0.8160	8.04E−02	−0.1305	6.47E−01	−0.1982	4.48E−01	0.98 (0.96, 1.01)	2.07E−01	0.99 (0.97, 1.01)	2.72E−01
LRRC10B_1	1.4129	2.41E−01	1.8385	7.68E−02	1.2674	4.38E−02	1.2944	2.57E−02	1.04 (0.99, 1.10)	1.19E−01	1.05 (1.00, 1.10)	3.85E−02
LRRC10B_2	1.7959	3.63E−01	−0.1150	9.46E−01	0.0736	9.43E−01	−0.4465	6.41E−01	1.05 (0.97, 1.15)	2.46E−01	1.01 (0.93, 1.09)	8.31E−01
PDE3A_1	0.2955	4.68E−01	0.4486	2.07E−01	0.4658	2.82E−02	0.4389	2.68E−02	1.01 (0.99, 1.03)	3.60E−01	1.01 (0.99, 1.03)	2.65E−01
PDE3A_2	−0.7590	8.36E−02	−0.3259	3.91E−01	0.0164	9.43E−01	0.0678	7.50E−01	0.98 (0.96, 1.00)	6.53E−02	0.99 (0.97, 1.01)	2.06E−01
PDE3A_3	0.3556	5.39E−01	0.3277	5.12E−01	0.7099	1.88E−02	0.5743	3.97E−02	1.01 (0.98, 1.03)	5.95E−01	1.00 (0.98, 1.03)	7.15E−01
PRDM6_2	−2.9031	**1.37E−04**	−2.2592	**6.10E−04**	−1.0295	2.40E−02	−0.8321	4.70E−02	0.94 (0.91, 0.97)	**1.83E−04**	0.95 (0.92, 0.98)	**7.71E−04**
PRDM6_3	−0.0133	9.84E−01	0.0288	9.60E−01	0.5288	1.33E−01	0.4501	1.65E−01	1.00 (0.97, 1.03)	9.68E−01	1.00 (0.97, 1.02)	9.00E−01
PRDM6_4	2.8922	2.27E−02	1.6951	1.24E−01	2.7579	**3.20E−05**	2.0433	**9.00E−04**	1.07 (1.01, 1.13)	2.48E−02	1.03 (0.98, 1.08)	2.18E−01
PRDM6_5	0.8160	8.05E−02	0.6358	1.15E−01	0.7446	**2.24E−03**	0.6116	6.57E−03	1.02 (1.00, 1.04)	3.18E−02	1.02 (1.00,1.04)	6.65E−02
SYT7_1	2.3106	4.88E−02	2.0600	4.23E−02	2.0556	**7.96E−04**	1.8727	**9.58E−04**	1.06 (1.00, 1.11)	3.56E−02	1.05 (1.00, 1.10)	4.36E−02
TBX2_1	−0.2606	7.29E−02	−0.0849	4.97E−01	−0.0632	4.05E−01	−0.0144	8.36E−01	0.99 (0.99, 1.00)	2.48E−02	1.00 (0.99, 1.00)	1.88E−01
TBX2_2	−0.6733	2.89E−02	−0.3934	1.38E−01	−0.1799	2.64E−01	−0.1090	4.62E−01	0.99 (0.98, 1.00)	1.26E−01	0.99 (0.98, 1.01)	3.67E−01
TBX2_3	1.1142	1.27E−01	0.5506	3.83E−01	1.1600	**2.33E−03**	0.9897	**4.99E−03**	1.02 (0.98, 1.05)	3.59E−01	1.00 (0.97, 1.03)	8.48E−01
TTBK1	−0.5550	2.07E−01	−0.6474	8.88E−02	−0.0657	7.75E−01	−0.1076	6.13E−01	0.99 (0.97, 1.01)	2.07E−01	0.99 (0.97, 1.00)	9.06E−02

CI: confidence interval; DBP: diastolic blood pressure; OR: odds ratio; SBP: systolic blood pressure.

a Adjusted for age, BMI, TC, TG, HDL−C, LDL−C, and FBG. *p* < 5.0 × 10^–−3^ were highlighted in bold.

**TABLE 4:** The associations between methylation levels and BP and hypertension.

Targets	Traits	Model 1^a^		Model 2^a^		Model 3^a^		Model 4^a^	
		Beta	*p* Value	Beta	*p* Value	Beta	*p* Value	Beta	*p* Value
C17orf82_3	DBP	1.0403	5.24E−04	0.9879	5.05E−04	0.9742	5.44E−04	0.9930	3.24E−04
HDAC9_1	DBP	1.1825	8.60E−05	1.1007	8.02E−05	0.8543	1.16E−03	0.8770	1.67E−03
IGFBP3_2	DBP	0.9563	2.59E−04	0.8552	4.26E−04	0.8343	5.58E−03	0.7357	2.30E−03
PRDM6_4	DBP	2.7579	3.20E−05	2.5434	3.53E−05	2.0314	8.74E−04	2.0433	9.00E−04
SYT7_1	DBP	2.0556	7.96E−04	1.9585	5.66E−04	1.9193	1.29E−04	1.8727	9.58E−04
PRDM6_2	SBP	−2.9031	1.37E−04	−2.1230	7.48E−04	−2.0581	7.64E−04	−2.2592	6.10E−04
PRDM6_2	HTN	−0.0573	1.83E−04	−0.0570	3.40E−04	−0.0550	2.98E−04	−0.0450	7.71E−04

CI: confidence interval; DBP: diastolic blood pressure; HTN: hypertension; OR: odds ratio; SBP: systolic blood pressure.

a Model 1 was an unadjusted regression model; Model 2 adjusted for age; Model 3 adjusted for age and BMI; Model 4 adjusted for age, BMI, TC, TG, HDL−C, LDL−C, and FBG.

### Association Between Methylation Levels and Hypertension

**FIGURE 3:**
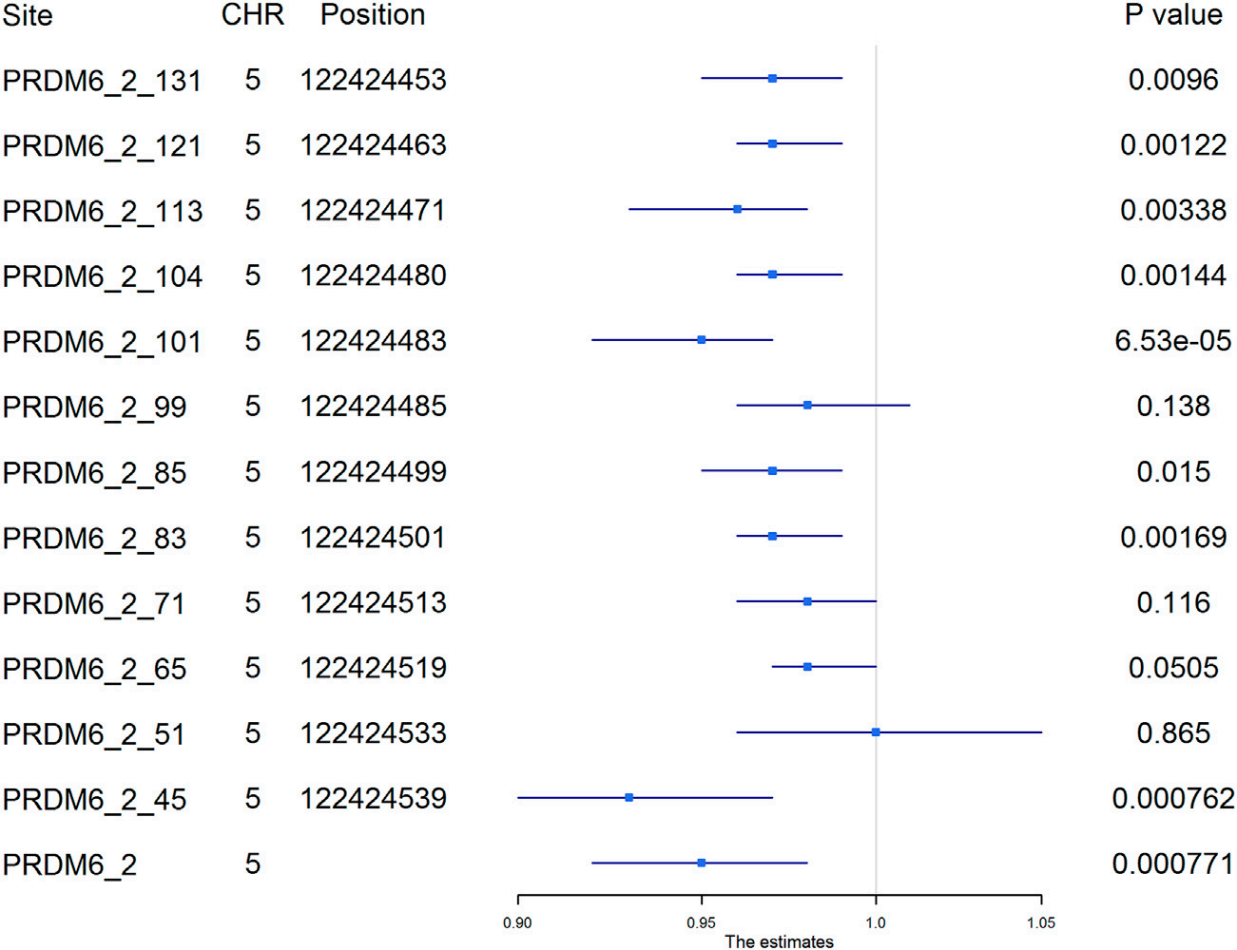
The association between PRDM6_2 methylation and hypertension. The plot presented the ORs and the corresponding 95% CIs for the estimations of the effects of *PRDM6* gene methylation on hypertension risk after adjusting for age, sex, smoking, drinking, body mass index, total cholesterol, and glucose. Methylation levels of eight sites in this gene were nominally associated with hypertension (adjusted *p* < 0.05). High methylation levels in these sites were associated with a lower risk of hypertension. The names of the methylation sites were defined by “gene name_target number_ position of the site in the target”. The genomic position assembly was hg19.

Among the 1,241 individuals, 485 (39.0%) were defined as hypertensive cases (Table 1). The proportion of males, smokers and drinkers were not significantly different between cases and controls. Compared with the controls, the mean age, SBP, DBP, BMI, TC, LDL-C, TG and FBG levels were significantly higher in hypertensive patients. The mean HDL-C levels were lower in hypertensive patients. Methylation levels of 13 sites were associated with hypertension after adjusting for covariates (FDR < 0.05) (Supplementary Table S6). Methylation levels of three sites in *PRDM6* and three sites in *SYT7* were significantly associated with hypertension (*p* < 1.0 × 10^–4^) (Figure 2). Higher methylation levels of sites in target PRDM6_2 were associated with lower hypertension risk, while higher methylation levels of sites in target SYT7_1 were associated with higher hypertension risk. At the target level, only methylation of PRDM6_2 was significantly associated with hypertension, with an OR of 0.95 (adjusting *p* = 7.71 × 10^–4^) (Table 3). In this target, methylation levels of eight sites were nominally associated with hypertension risk (adjusted *p* < 0.05) (Figure 3). The adjusted ORs for these eight sites ranged from 0.93 to 0.97. Therefore, among the test DNA methylations, methylations in *PRDM6* were significantly associated with both DBP and hypertension (Table 3).

### Association Between *IGFBP3* Methylation Levels and BP in Europeans

**FIGURE 4:**
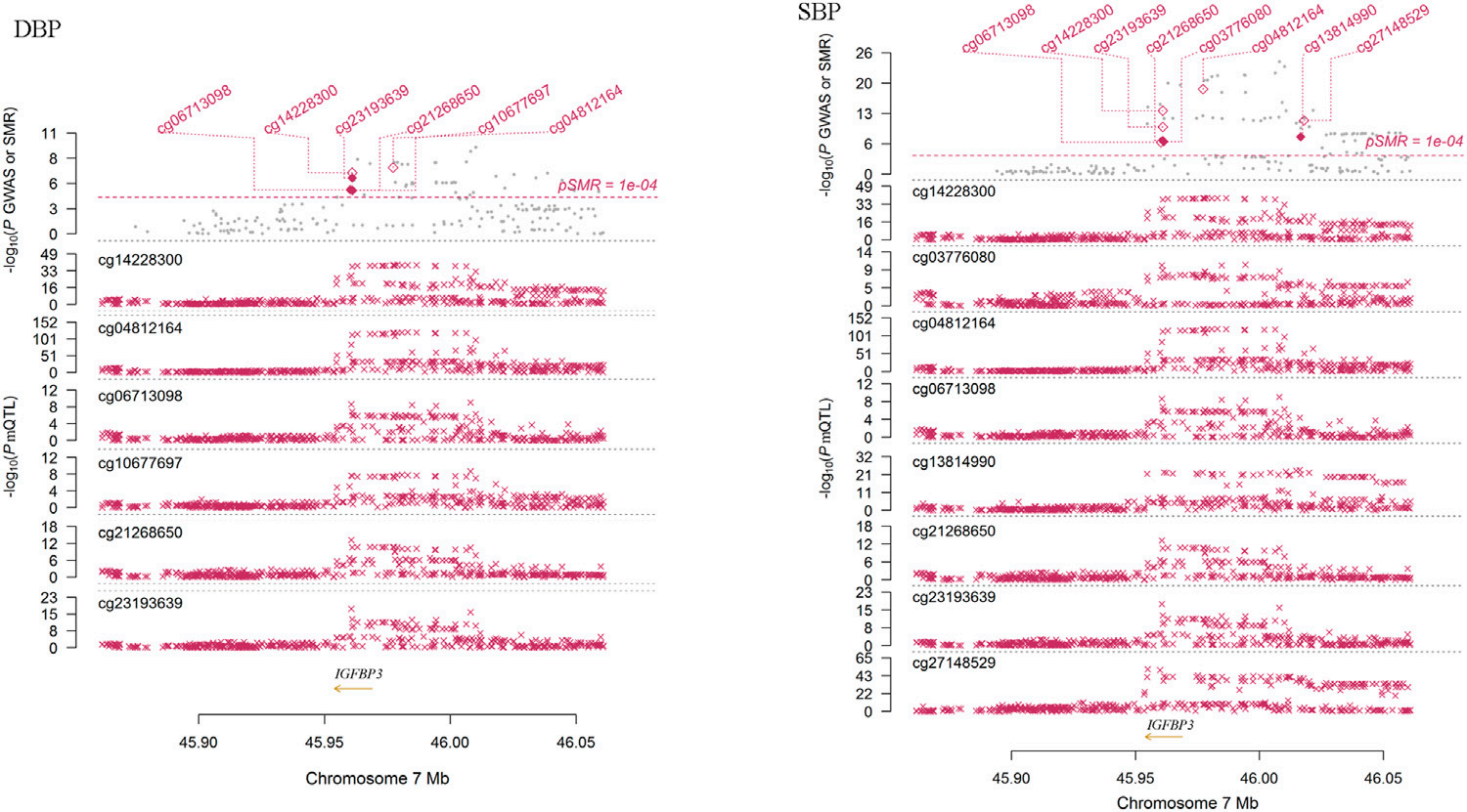
The association between *IGFBP3* methylation and DBP in Europeans. This figure presented the result of SMR analysis. The *x*-axis represented the genomic position (GRCh37.p13). Along the *y*-axis direction the figure consisted of two parts. The lower part showed the information of mQTL for six methylation sites. The *y*-axis represents -log10 (*P* mQTL). In this part, we can see that *IGFBP3* SNPs were strongly associated with *IGFBP3* methylation levels. The upper part shows the results of GWAS and SMR analysis. The *y*-axis represents −log_10_ (*p* value of GWAS or SMR). In this part, we found that *IGFBP3* SNPs were strongly associated with DBP according to the GWAS (grey dots) and *IGFBP3* methylation levels were associated with DBP (diamonds).

We performed SMR analysis to find additional evidence on the pleiotropic association between methylations of the tested genes and BP. As shown in Figure 4, the methylation levels of six sites (cg06713098, cg14228300, cg23193639, cg21268650, cg10677697 and cg04812164) around *IGFBP3* were significantly associated with DBP in European populations (*P*_SMR_ = 1.58 × 10^–5^, 2.15 × 10^–7^, 8.19 × 10^–7^, 1.78 × 10^–5^, 1.88 × 10^–5^ and 5.84 × 10^–8^, *P*_HEIDI_ = 0.524, 0.058, 0.379, 0.804, 0.621 and 0.048, respectively). Genetic variants associated with DBP identified by GWAS were strongly associated with the methylations. In *IGFBP3* gene, eight CpG sites were found to be associated with SBP (Figure 4). Among them, four sites (cg14228300, cg21268650, cg03776080 and cg13814990) passed the HEIDI test (*P*_SMR_ = 2.68 × 10^–14^, 6.85 × 10^–8^, 1.33 × 10^–7^ and 1.15 × 10^–8^, *P*_HEIDI_ = 0.926, 0.238, 0.445 and 0.994, respectively). In addition to these sites in *IGFBP3*, methylation levels of CpG sites in *PRDM6* (cg23290100, cg16368670 and cg16069012), *HDAC9* (cg16925459) and *SYT7* (cg00009053 and cg17683593) were associated with BP (*P*_SMR_ *<* 5.0 × 10^–6^), but only the associations between cg16925459 methylation level and DBP passed the HEIDI test (*P*_SMR_ = 2.07 × 10^–6^, *P*_HEIDI_ = 0.087).

We further evaluated the causal relationships between methylation levels of cg06713098, cg14228300, cg23193639, cg21268650, cg10677697 and cg04812164 and BP using several other MR methods. The threshold for instrumental variable selection was set to 5.0 × 10^–4^. After LD clumping we obtained enough independent instrumental variables for cg06713098, cg14228300, cg23193639 and cg04812164. Therefore, the associations between these four sites and DBP and SBP were examined by 4 MR methods (Table 5). By using the IVW method, these four sites were all significantly associated with BP. By using the MR-Egger method, methylation levels of cg06713098, cg14228300 and cg04812164 were associated with DBP and SBP, but the associations between cg14228300 and DBP were not significant. By using the MRPRESSO method, the associations between cg23193639 and SBP was not significant. In CAUSE analysis, methylation level of cg14228300 was significantly associated with SBP (*p* = 1.02 × 10^–3^). The association between cg14228300 and cg04812164 and SBP and the association between cg14228300 and cg06713098 and DBP were validated by 4 MR methods.

**TABLE 5:** *p* values of associations between the four CpG sites in *IGFBP3* and BP in different Mendelian randomization analysis.

Traits	CpG sites	IVW	MR−Egger	MRPRESSO	CAUSE
DBP	cg06713098	1.30E−06	2.06E−04	8.40E−03	1.50E−02
DBP	cg14228300	5.55E−10	5.60E−02	6.69E−05	4.83E−02
DBP	cg23193639	8.87E−04	3.37E−02	4.00E−03	2.40E−01
DBP	cg04812164	9.51E−08	2.63E−06	2.27E−05	1.86E−01
SBP	cg06713098	3.39E−03	1.06E−03	2.91E−02	1.26E−01
SBP	cg14228300	6.78E−29	4.07E−05	2.46E−07	1.02E−03
SBP	cg23193639	6.06E−03	6.06E−03	1.76E−01	5.68E−01
SBP	cg04812164	3.10E−15	4.60E−13	1.03E−07	4.67E−02

**FIGURE 5:**
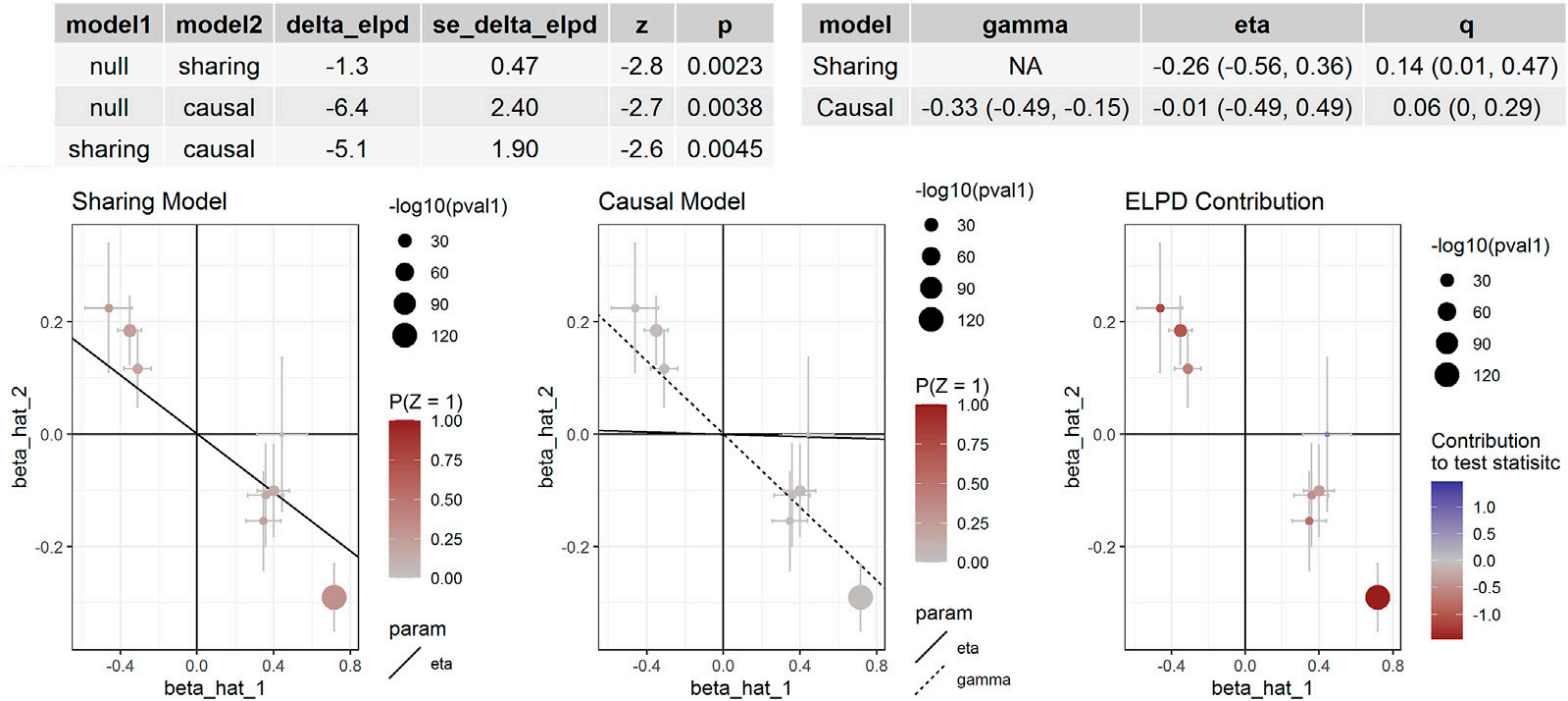
The association between cg04812164 methylation and SBP in Europeans. This figure presented the results of the CAUSE analysis for the association between cg04812164 methylation and SBP. The analysis showed that the causal model was significantly better than both the null and the sharing models, indicating the causal relationship between cg04812164 and SBP.

If the threshold for instrumental variable selection was set to 5.0 × 10^–8^, 3 SNPs for cg14228300 and 9 SNPs for cg04812164 were retained. By using these instrumental variables we found significant associations between these sites and SBP and DBP, except for the association between cg04812164 and DBP in the CAUSE analysis (*p* = 0.057). The IVW, MR-Egger and CAUSE methods all indicated causal association for cg14228300 with SBP. MRPRESSO could not run because 3 instrumental variables were not enough for the method. The IVW, MR-Egger, MRPRESSO and CAUSE methods all indicated causal association for cg04812164 with SBP using 9 instrumental variables. In CAUSE analysis, the causal model for the association between cg04812164 and SBP was better than the null model and the sharing model (Figure 5), indicating that cg04812164 may be causally associated with SBP.

### Association Between *IGFBP3* mRNA Levels and BP in Europeans

It is known that DNA methylation controls gene expression. If methylation functionally influences BP regulation, it may regulate BP by influencing gene expression. Therefore, we performed SMR analysis to find additional evidence on the pleiotropic association between the tested genes and BP at mRNA expression levels. We found that *IGFBP3* SNPs were strongly associated with *IGFBP3* mRNA level [eQTL (Westra et al., 2013)] and were strongly associated with DBP according to the GWAS. In SMR analysis, the mRNA levels of two probes (ILMN_2396875 and ILMN_1746085) for *IGFBP3* were associated with DBP (*P*_SMR_ = 7.94 × 10^–4^ and 3.12 × 10^–5^, respectively) (Supplementary Figure S1). However, the association did not pass the HEIDI test (*P*_HEIDI_ = 3.64 × 10^–4^ and 1.97 × 10^–8^, respectively). Similar results of associations between these two probes and SBP were found (Supplementary Figure S2). In IVW, MR-Egger, MRPRESSO and CAUSE analysis, we did not find significant associations between these probes and BP. It seems that the associations between mRNA expression levels and BP may be pleiotropic associations.

## Discussion

This study represents an effort to identify potential functional factors for BP inside genes identified by GWAS. We first conducted a cross-sectional study to examine associations between DNA methylations in the promoters of these genes and BP. We found that DNA methylations in the promoters of *PRDM6*, *HDAC9*, *IGFBP3*, *SYT7*, *TBX2* and *C17orf82* were significantly associated with BP or hypertension in the Chinese Han population. Yet it is important to recognize that correlation does not prove causality. Studies have demonstrated a causal role of DNA methylations in the regulation of BP (Richard et al., 2017). MR is an instrumental variable analysis approach which can reduce confounding or reverse causality. Therefore, MR analysis was then applied to find supporting evidence for causal associations. By MR analysis based on public summary statistics, we confirmed the associations of *IGFBP3* gene methylation and expression levels with BP. This is the first study to report that promoter DNA methylation levels of these genes were associated with BP and hypertension.

As suggested by previous studies, blood is utilized as a surrogate tissue for the study of more pathologically relevant tissues involved in cardiovascular disease, such as biopsies of the myocardium or of blood vessel walls (Kato et al., 2015; Rask-Andersen et al., 2016). Studies have shown that blood DNA methylation plays important roles in human atherosclerosis (Baccarelli et al., 2010; Zaina et al., 2014; Rask-Andersen et al., 2016; Nakatochi et al., 2017), and a few studies have been performed to identify epigenetic risk factors for stroke (Baccarelli et al., 2010; Rask-Andersen et al., 2016; Soriano-Tarraga et al., 2016). Hypertension is one of the most important risk factors for cardiovascular diseases and shares many genetic and environmental risk factors with cardiovascular diseases. Studies have suggested that heritable DNA methylation plays a role in regulating BP (Richard et al., 2017). In Richard et al.’s study, MR analysis suggested the presence of causal regulatory relations among select methylation sites, BP, and gene expression (Richard et al., 2017). Studies have also suggested that the association between DNA methylation and BP might be different between different populations (Lewington et al., 2016). Among the 10 genes that were suggested to be related to BP by GWAS, we showed that methylation levels of *PRDM6*, *HDAC9*, *IGFBP3*, *SYT7*, *PDE3A*, *TBX2* and *C17orf82* in blood cells were associated with BP and that methylation levels of *PRDM6* and *SYT7* were associated with hypertension risk among Chinese individuals. None of these associations have been reported in previous studies. We also performed MR analysis to find supporting evidence for causal associations and finally showed that *IGFBP3* gene methylation in blood cells may be causally associated with BP, and the associations may not be due to linkage or pleiotropy according to the results of several MR methods, especially the CAUSE method, which accounts for correlated and uncorrelated pleiotropic effects. These findings added new epigenetic risk factors for hypertension.

We noticed that most of the newly identified CpG sites overlapped with promoters or enhancers. Moreover, the genomic region of these CpG sites have long-range interactions with nearby promoters or enhancers. For example, the significant methylation sites in target PRDM6_2 were located in a CpG island (chr5:122424338–122424539, CpG count: 21). This CpG island overlaps a promoter (chr5:122424113–122425192) at the 5′ regions of *PRDM6*. The genomic region of this promoter interacts with two nearby enhancers (chr5:122433360–122436028 and chr5:122454855–122455750). The protein (a putative histone-lysine N-Methyltransferase) encoded by *PRDM6* is involved in the regulation of contractile proteins in vascular smooth muscle cells (Davis et al., 2006). *PRDM6* variants were associated with cardiac structure and function (Vasan et al., 2009), and glucose and insulin traits (Meigs et al., 2007). The present study showed that *PRDM6* methylations were associated with BP and hypertension. Therefore, it seems that the identified DNA methylations may have a potential of gene expression regulation, and may play a functional role in hypertension.

Besides, *IGFBP3* (insulin like growth factor binding protein 3) is a well-known cardiovascular disease susceptibility gene. The tested targets were located in a CpG island (chr7:45960138-45961347, CpG count: 139). This CpG island overlaps a promoter (chr7:45957891-45964736) at the 5′ regions of *IGFBP3*. Previous study has identified causal methylations for BP by MR approach (Richard et al., 2017). The present study first showed the significant causal association between *IGFBP3* methylation and BP by applying several MR approaches. Among the six sites identified by MR analysis, cg06713098, cg14228300 and cg23193639 were located in the tested CpG island (chr7:45960138-45961347). The site cg06713098 was tested and analyzed in the present observational study (site IGFBP3_2_113), and was significantly associated with DBP. Thus, the association between cg06713098 and DBP were confirmed in both observational study and MR analysis. Besides, suggested association between mRNA level of *IGFBP3* in blood cells and DBP was also found. Therefore, promoter methylations in *IGFBP3* may be functional BP regulatory element. These methylations may be involved in the regulatory pathway linking genetic variants to BP. Circulating level of IGFBP3 has been shown to be associated with hypertension, stroke, carotid atherosclerosis and several cardiovascular disease risk factors (Schwab et al., 1997; Watanabe et al., 2003; Kawachi et al., 2005; Capoluongo et al., 2006; Kaplan et al., 2007; Lam et al., 2010; Ebinger et al., 2015). But by applying MR analysis we did not find a causal association between circulating IGFBP3 levels and BP. Further studies may need to examine the association of blood cell IGFBP3 protein levels with BP.

The other BP-associated genes have been shown to play roles in cardiovascular diseases. *TBX2* encodes transcription factors involved in the regulation of developmental processes. *TBX2* variants were associated with kidney function and chronic kidney disease (Chambers et al., 2010; Kottgen et al., 2010). Genetic variants in the *C17orf82* gene region were associated with serum urate concentrations (Kottgen et al., 2013) and chronic kidney disease (Kottgen et al., 2010). *HDAC9* encodes histone deacetylase 9. *HDAC9* genetic variants were associated with stroke (Malik et al., 2018) and mediate their effects through increased *HDAC9* expression and increase ischemic stroke risk via promoting carotid atherosclerosis (Markus et al., 2013; Azghandi et al., 2015). The BP-associated genetic locus rs2023938 of *HDAC9* has been shown to overlap myocardial infarction-associated CpG sites (Rask-Andersen et al., 2016). SYT7 (Synaptotagmin 7) acts as a regulator of Ca (2+)-dependent insulin and glucagon secretion in beta-cells (Gustavsson et al., 2009; Dolai et al., 2016). The role of this gene in cardiovascular diseases is unclear either. According to the findings of a previous study and the present study, methylations in these genes may be potential functional elements that affect gene expression and may be potential risk factor for hypertension.

Some limitations of this study should be mentioned. First, this is not a matched case-control design study but a cross-sectional study, in which several covariates were significantly different between hypertension cases and control. Second, although we have obtained significant findings in this study, we only sequenced a small fragment of the genomic region of the genes. To comprehensively evaluate the effects of methylations on BP, whole genomic region of the CpG Islands are suggested to sequence in large samples. Third, the MR analysis only examined the association between *IGFBP3* methylation levels and BP. The other genes were not examined due to lacking in data. In addition, the number of instrumental variables for some of the MR analyses is very limited, which might affect the reliability of the MR analysis.

## Conclusion

In summary, the present study showed that DNA methylation in the promoters of *PRDM6*, *HDAC9*, *IGFBP3*, *LRRC10B*, *SYT7*, *PDE3A*, *TBX2* and *C17orf82* genes were significantly associated with BP or hypertension. These promoter DNA methylations probably play functional roles in BP regulation. This study increases our understanding of the role of GWAS-identified genes in the pathogenesis of hypertension. The associated methylations may be important regulatory elements and can be suggested as important candidates for further functional studies. Although we detected significant associations, further functional studies are needed to elucidate the mechanisms.

## Data Availability

The original contributions presented in the study are included in the article/Supplementary Materials, further inquiries can be directed to the corresponding authors.
