# Supplementary material for: Promoter DNA Methylation in GWAS-Identified Genes as Potential Functional Elements for Blood Pressure: An Observational and Mendelian Randomization Study
Source: Front Genet. 2022 Jan 11;12:791146. doi: 10.3389/fgene.2021.791146 (PMC8787193; doi:10.3389/fgene.2021.791146)
Supplement: Supplementary file 1 [file DataSheet2.docx]

**
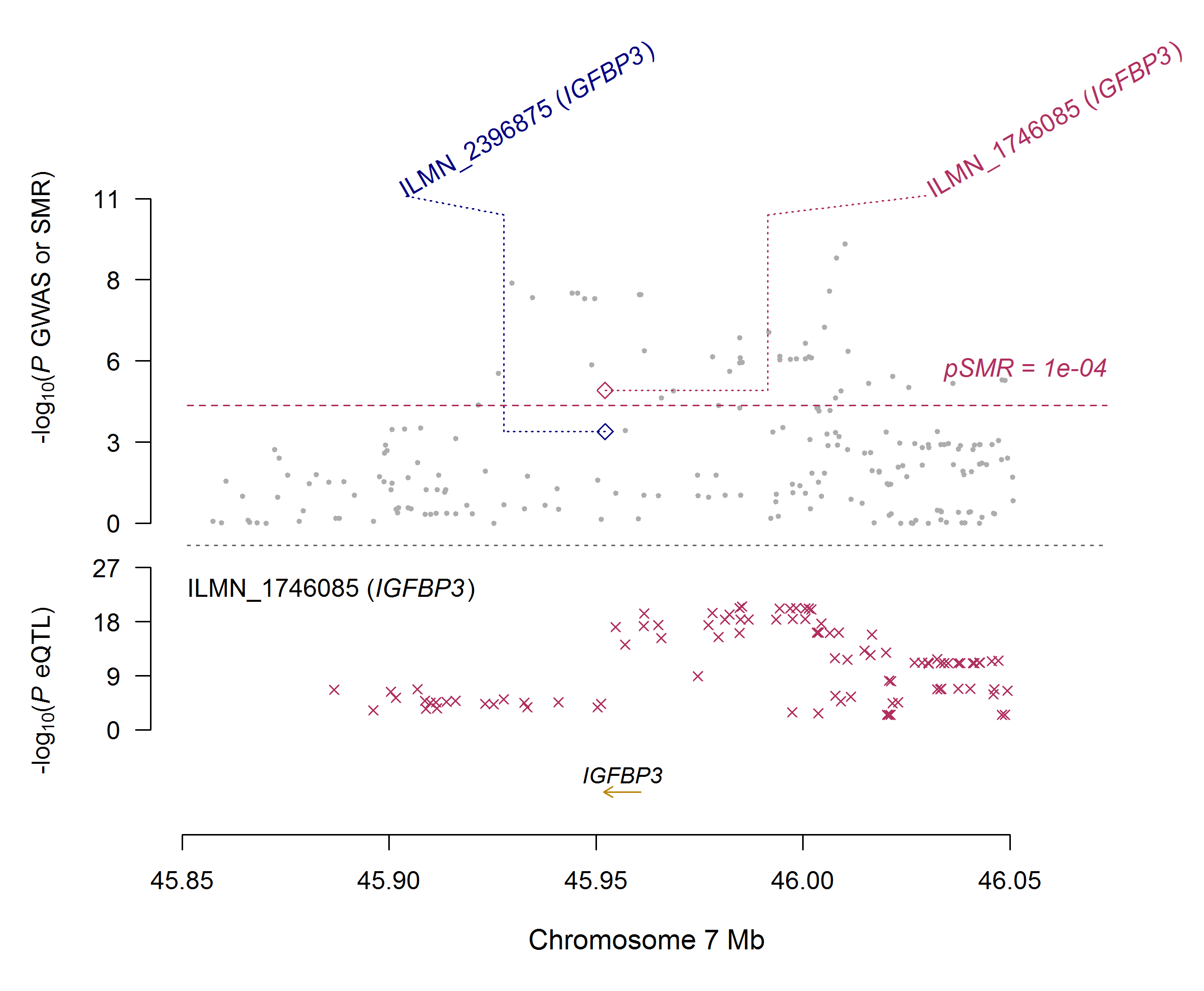
**

**Supplementary figure S1 The association between *IGFBP3* mRNA levels and DBP in Europeans.**

The figure consists of 2 parts. The x-axis represents the genomic position (GRCh37.p13). The lower part of the figure shows the results of eQTL. The y-axis represents -log10 (*P* eQTL). In this part, we can see that *IGFBP3* SNPs were strongly associated with *IGFBP3* mRNA level (eQTL, Westra et al. 2013 Nat Genet). The upper part shows the results of GWAS and SMR analysis. The y-axis represents -log_10_ (*P* value of GWAS or SMR). In this part, we found that *IGFBP3* SNPs were strongly associated with DBP according to the GWAS (grey dots) and *IGFBP3* mRNA levels were associated with DBP (diamonds).


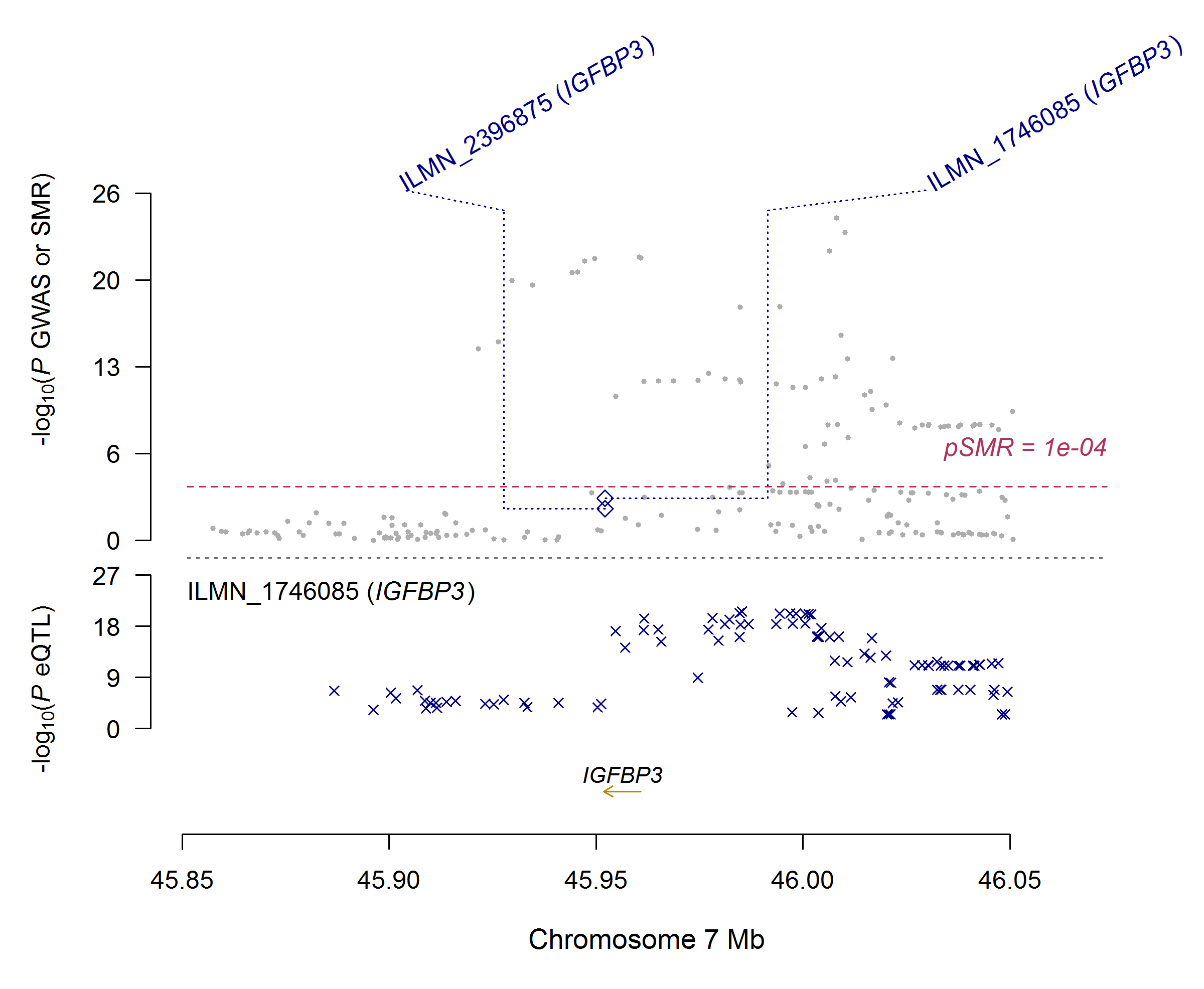


**Supplementary figure S2 The association between *IGFBP3* mRNA levels and SBP in Europeans.**

The figure consists of 2 parts. The x-axis represents the genomic position (GRCh37.p13). The lower part of the figure shows the results of eQTL. The y-axis represents -log10 (*P* eQTL). In this part, we can see that *IGFBP3* SNPs were strongly associated with *IGFBP3* mRNA level (eQTL, Westra et al. 2013 Nat Genet). The upper part shows the results of GWAS and SMR analysis. The y-axis represents -log_10_ (*P* value of GWAS or SMR). In this part, we found that *IGFBP3* SNPs were strongly associated with SBP according to the GWAS (grey dots) and *IGFBP3* mRNA levels were associated with SBP (diamonds).
